# School workers’ knowledge, attitude and behaviour related to use of Toombak: a cross sectional study from Khartoum state, Sudan

**DOI:** 10.1186/s12903-017-0460-8

**Published:** 2017-12-21

**Authors:** Hatim Mohammed Almahdi, Anne Nordrehaug Åstrøm, Raouf Wahab Ali, Elwalid Fadul Nasir

**Affiliations:** 1 University of Science and Technology, Faculty of Dentistry, Omdurman, Sudan; 2 Centre for International Health, University of Bergen, Bergen, Norway; 3 Department of Clinical Dentistry, Faculty of Medicine and Odontology, University of Bergen, Bergen, Norway; 4 Centre for Oral Health Expertise, Western-Hordaland, Bergen, Norway

**Keywords:** Toombak, Smokeless tobacco SLT, School workers, Knowledge, Attitude, Sudan

## Abstract

**Background:**

Toombak is a form of smokeless tobacco (SLT) that is locally made and consumed in Sudan and contains several carcinogenic elements. Use of Toombak has been etiologically linked to various oral diseases including oral cancer. This study aimed to obtain baseline information about the Toombak use among Sudanese school workers, as well as their knowledge about Toombak related health hazards and attitude towards their role in Toombak control. In addition, this study assessed the availability and effectiveness of control policies and preventive practices in the schools.

**Methods:**

A cross-sectional school-based study using one-stage stratified random sampling procedure; four schools were selected randomly from each of seven localities. A total of 239 school workers’ were recruited (census) from the selected schools in Khartoum State, Sudan.

**Results:**

Of the school workers, 63% (147) were ≤40 years, half were females and 79.2% (187) were teachers. A total of 9.6% (22) school workers confirmed ever use of Toombak and the percentage of daily users amounted to 64.7% (11). Moreover, 76.2% (16) of ever Toombak users were ≥40 years and all of them were males (*p* < 0.001). Most of the school workers reported good knowledge, positive attitude towards their role in Toombak control and good preventive practice. Female school workers were more likely to report positive attitude towards their role in Toombak control (*p* < 05), and to report good knowledge. Those reporting good preventive practice in schools reported good knowledge more than two times than their counterpart (*p* < 0.001). Age was the strongest predictor of ever Toombak use among school workers (*p* < .001).

**Conclusions:**

The use of Toombak among school workers was associated with poor knowledge, negative attitude towards their role in Toombak control and poor preventive practice, Therefore, school workers use of Toombak may reduce their motivation and use of their potential in the prevention of a major health problem caused by Toombak use and affects their role model behaviour. On the other hand, school workers engagement with preventive practices in schools’ was associated with good knowledge which in turn empowers their positive attitude towards their role in Toombak control.

## Background

The tobacco epidemic is a major public health problem and one of the main causes of death and disability worldwide [1]. More than 300 million people in at least 70 countries, use smokeless tobacco (SLT) [2]. Toombak is a form of SLT that is locally made and consumed in Sudan with the prevalence of 34% among adults [3]. Chemical analysis of Toombak has revealed that it contains at least 100-fold higher concentrations of the Tobacco specific N-nitrosamines (TSNAs) than the commercial SLT brands from US and Sweden [4]. These substances have also been reported to be responsible for potentially malignant oral lesions and oral cancer [5, 6].

Use of Toombak has been etiologically linked to various oral diseases, such as periodontal diseases, mucosal lesions and may eventually lead to tooth loss [7, 8]. Moreover, the etiologic link between use of Toombak and oral cancer in Sudan has been confirmed in several studies [9–15].

School workers (teachers and other staff-members; labourers, clerical and administrators) represent important role models for secondary school students. The daily interactions between students and school workers combined with their authority, make them a potentially influential group with respect to Toombak use control [16, 17]. Therefore, school workers play a vital role in influencing the students during adolescence which is a period in life when many young people are starting to use tobacco products [16]. School workers can advice about the harmful health effects of Toombak use, as well as how to quit Toombak related behaviour. Therefore, utilisation and training of school workers should be a crucial part of tobacco prevention programs as recommended by the World Health Organization (WHO) Framework Convention on Tobacco Control (FCTC) [15]. Use of school workers as role models for students provides opportunities to integrate tobacco control and prevention programs with school education. Implementation of schools’ tobacco control policy, on the other hand, is an effective factor which has a major impact on students and has been associated with lower use of tobacco [17–19].

Khartoum is the capital of the Sudan and consists of three major cities; Khartoum, Omdurman and Khartoum North (Bahry). The total population of the Republic of the Sudan is 36 million people, where Khartoum state has a population of 6 million people [20].

In Sudan, estimates of the prevalence and correlates of Toombak use are scarce. Research on Toombak is important and relevant regarding the magnitude and impact of the problem. This study aimed to assess baseline information about Toombak use among Sudanese school workers, their knowledge about Toombak related health hazards and their attitude towards their role in Toombak control. In addition, the study aimed to assess the availability and effectiveness of control policies and the preventive practices in the schools. Such data are necessary for the planning and implementation of intervention programs to prevent Toombak use among school workers.

## Methods

This cross-sectional school-based study was carried out during the period (2013–2014) as a part of a larger research project focusing on the use of Toombak among secondary school students in Khartoum State, Sudan.

### Sampling procedures

The sampling procedure was made for a larger-scale study of secondary schools students’ use of Toombak [21].

The three cities and their respective localities were all represented in the sample a total of 28 schools; four schools from each locality (public/female, public/male, private/female and private/male) were randomly selected with the substitution of the schools which did not agree to participate. All school workers (census) in the 28 selected schools were invited to participate in the study. Eligibility criteria required was the presence of the school workers at the time of data collection. A total of 239 school workers (teachers and other staff-members; labourers, clerical and administrators) were invited and accepted to participate in the study from the 28 previously selected secondary schools.

### Data collection

School workers completed self-administered questionnaires at their offices. The data collection was supervised by trained personnel (the main researcher and two assistants). After a brief explanation of the objectives of the study, written informed consent was obtained from the participants.

The questionnaire, used in this study was based on the Global Schools’ Personnel Survey (GSPS) questionnaire; GSPS is an integral part of the Global Tobacco Surveillance System (GTSS), (SLT section questions) started by the WHO in 1999 [22]. The questionnaire was administered in Arabic to collect information on Toombak use, knowledge about Toombak related health hazards and perception of school workers towards their role in Toombak control, as well as the availability and effectiveness of Toombak control policies, and preventive practices in schools. The translation from English to Arabic and vice versa was done by experts in both languages and piloted to test for the accuracy of translation and understanding of the questions before administration in the schools. The pilot was conducted in two schools (male, female) including 24 school workers. The needed adjustments were performed for the final questionnaire.

### Questions and variables

*Ever use of Toombak* was measured by the question; *“have you ever used Toombak”* using response options (1) “yes”; (2) “no”. The original categories recoded into (0) non- user (includes original response 2); (1) ever user (includes original response 1). *The frequency of Toombak use* was measured by the question; “*how frequently do you use Toombak at present*” using response options (1)” daily”; (2) “occasionally”.

*Age group* was measured by the question; “*how old are you*” using response options (1) “19 years or younger”; (2) “20 to 29 years”; (3) “30 to 39 years”; (4) “40 to 49 years”; (5) 50 to 59 years; (6) “60 years old or older”. The original categories recoded into (0) < 40 years (including original categories 1, 2, 3); (1) ≥ 40 years (including original categories 4, 5, 6).

*Knowledge about Toombak related health hazards* was measured by four questions; “*is Toombak use addictive*”; “*does Toombak use cause oral cancer*”; “*does Toombak use cause heart disease*”; using response options (1) “yes”; (2) “no”; (3) “I don’t know”. The original categories recoded into (0) poor knowledge (includes original responses 2, 3); (1) good knowledge (include original response 1); “*does Toombak use cause malaria*” using response options (1) “yes”; (2) “no”; (3) “I don’t know”. The original categories recoded into (0) poor knowledge (includes original responses 1, 3); (1) good knowledge (include original response 2). A sum score was constructed, labelled “*knowledge about Toombak related health hazards*” from the four questions (0–4) Cronbach’s α =0.45). For analysis, this sum score was dichotomized on the median split (median 3, IQR 1)with values (0) poor knowledge (initial scores 0, 1, 2); (1) good knowledge (initial scores 3, 4).

*Attitude of school workers towards their role in Toombak* control was measured by four questions; “*school workers’ use of Toombak influences students use*”; *“schools should have a policy or rule specifically prohibiting Toombak use among students on school premises/property”; “Have you ever advised a student to stop using or quit use of Toombak”;* using the response options (1)” yes”; (2) “no”; (3) “I don’t know”; the original categories were recoded into (0) negative attitude (original categories 2, 3); (1) positive attitude (original categories 1). “*How concerned are you about Toombak use among youth in your community*”; using response (1) “very concerned”; (2) “Somewhat concerned”; (3) “Not at all concerned”. Original categories were recoded into (0) negative attitude (original response 3); (1) positive attitude (includes original responses 1, 2). A sum variable was constructed ranging from (0–4), labelled “*Attitude of school workers towards their role in Toombak* control” (Cronbach’s α = 0.54) and dichotomized into (0) negative attitude (initial scores 0) (1) positive attitude (initial scores 1–4).

*Availability of a policy to prevent use of Toombak in schools* was measured by the question; “*does school has policy that prohibits Toombak use within school premises*” using the response options (1)” yes”; (2) “no”; (3) “I don’t know”; response options were recoded (0) no policy (original code 2, 3); (1) yes, there is policy (original code 1).

*Effectiveness of schools’ policy* was measured by the question “*does school policy prohibit Toombak for visitors, students, and school workers’*” using response options (1) “yes”; (2) “no”; (3) “I don’t know”; response options recoded (0) no effective policy (original codes 2, 3); (1) effective policy (original code 1).

*Preventive practices in school* were measured by five questions; “*did you participate in campaigns against Toombak use”;* “*is prevention of Toombak use included in the school curriculum*”; “*do you have access to teaching materials for Toombak prevention*”; “*have you received training to prevent Toombak use among students*” ; “*do you use non-classroom activities to teach Toombak prevention to students*” using response options (1) “yes”; (2) “no”; (3) “I don’t know”; response options were recoded into (0) poor practice (original code 2, 3); (1) good practice (original code 1). A sum score was created from five questions (0–5) and labelled “*preventive practices in schools*” (Cronbach’s α = 0.61). This sum score was dichotomized on the median split (median 2, IQR 2) into (0) poor practice (initial scores 0, 1); (1) good practice (initial scores 2, 3, 4, 5).

### Data analysis

Data were recorded and analysed using the Statistical Package for the Social Science, version 20 (IBM SPSS Statistics). Descriptive analyses were performed using frequencies and percentages. For the bivariate analysis, chi-square tests were performed to evaluate the categorical variables and the level of significance was set at *p* < 0.05. Multiple variable analysis was conducted using multiple logistic regression. Estimates were presented as odds ratios (OR) and 95% Confidence Intervals (CI). Nagelkerkes R^2^ was calculated for each multiple variable logistic regression model. Nagelkerkes R^2^ is a pseudo R square that generalize the coefficient of determination with values between 0and 1 where 0 denotes that the model for not explain anything about the variation in the dependent variable and 1 that the model completely explain the variance.

## Results

Most of the school workers 63.1% (147) were <40 years. Females represented 50.2% (114) of the participants. The majority 79.2% (187) were teachers, 7.2% (17) were headmasters, 1.7% cleric, 6.4% service labourers and 5.5% others. Almost half of the participating school workers 50% (116) confirmed that they teach health issues, while 64.3% (148) confirmed the availability of schools’ policy that prohibits Toombak use. Only 17% (38) reported that policies were completely enforced among school workers, whereas 35.1% (78) reported complete enforcement among students.

Almost half of the school workers, 55.3% (125), reported good knowledge with respect to health hazards related to using of Toombak, whereas 86.1% (192) had a positive attitude towards their role in Toombak control. About, 57.2% (127) confirmed good preventive practices in schools.

Prevalence of ever use of Toombak among school workers was 9.6% (22) among them 64.7% (11) were daily users and 35.3% (6) were occasional users. A 55% (11) reported using Toombak inside the school (*p* < 0.001). As depicted in Table 1, ever use of Toombak varied statistically significantly by age, gender.

**Table 1:** Percentage distribution of ever Toombak use by school workers demographic characteristics, knowledge, attitude, availability of school policies and preventive practices in schools

Characteristic	Ever use % (n)
Age group	
< 40 years	23.8 (5)
≥ 40 years	76.2 (16)^**^
Gender	
Female	0
Male	95 (21)^**^
Position	
Others	13.6 (3)
Teachers	86.4 (19)
Teaching health issues	
No	50 (11)
Yes	50 (11)
Knowledge about Toombak relate health hazards	
Poor knowledge	36.4 (8)
Good knowledge	63.6 (11)
Attitude towards their role in Toombak control	
negative attitude	36.4 (8)
positive attitude	63.6 (14)
Availability of the policy	
No	27.3 (6)
Yes	72.7 (16)
Effectiveness of schools’ policy	
No	33.3 (7)
Yes	66.7 (14)
Preventive practices in schools	
Poor practice	45.5 (10)
Good practice	54.5 (12)

^*^ *P* < 0.05, ^**^ *P* < 0.001

**Fig. 1:**
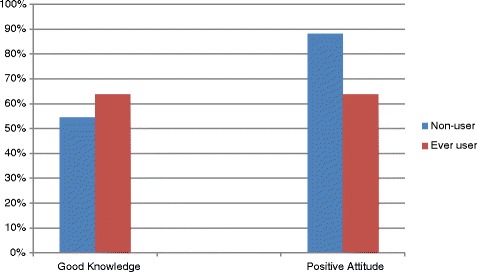
Demonstrates Good Knowledge about health hazards and Positive Attitudes of school workers towards their role in Toombak control, among ever users and non-Toombak users

As shown in Table 2, good knowledge regarding Toombak use related health hazards was significantly associated with teaching health issues (*p* < 0.05) and preventive practices in schools as those engaged in these practices tend to report good knowledge (*p* < 0.001).Whereas the larger proportion of female school workers reported positive attitude towards their role in Toombak control than males (*p* < 0.05). A significantly smaller proportion of ever Toombak users reported positive attitudes than did non-users (88.1% versus 63.6%) (Fig. 1).

**Table 2:** Percentage distribution of school workers good knowledge about health hazards and positive attitude by demographic characteristics, availability of school policies, preventive practices in schools and Toombak use

Characteristic	Good knowledge	Positive attitude
Age group	% (n)	% (n)
< 40 years	55. (77)	88.9 (120)
≥ 40 years	54.9 (45)	81.9 (68)
Gender		
Male	52.3 (57)	81.3 (87)
Female	58.5 (62)	92.5 (98)^*^
Position		
Others	66.7 (20)	75.9 (22)
Teachers	54.1 (105)	88 (169)
Teaching health issues		
No	47.2 (51)	86.5 (96)
Yes	62.5 (75)^*^	85 (91)
Knowledge about Toombak related health hazards		
Poor knowledge		84.2 (80)
Good knowledge		86.7 (104)
Availability of the policy		
No	51.3 (40)	80.8 (63)
Yes	57.1 (80)	88.3 (121)
Effectiveness of schools’ policy prohibit Toombak		
Not effective policy	53.4 (39)	84.4 (65)
Yes effective policy	58.4 (73)	85.7 (102)
Preventive practices		
Poor practice	43.3 (39)	85.9 (79)
Good practice	62.7 (79)^**^	85.8 (103)
Ever Toombak use		
Non-users	54.4 (106)	88.1 (170)
Ever user	63.6 (14)	63.6 (14)^**^

^*^ *P* < 0.05, ^**^ *P* < 0.001

Availability and effectiveness of the control policy in schools showed the discrepancy between the existence of the policies and its enforcement among students and school workers (Table 3).

**Table 3:** Percentages distribution % (n) of availability and effectiveness of control policy by ever Toombak user

Variable	% (n) non-users	Ever use % (n)	Total %(n)
Availability of policy prohibit Toombak			
No	92.6 (75)	7.4 (6)	100 (81)
Yes	88.7 (126)	11.3 (16)	100 (142)
Total	90.1 (201)	9.9 (22)	100 (223)
Effectiveness of policy for all			
Not effective	90.8 (69)	9.2(7)	100 (76)
Yes effective	89.1 (115)	10.9 (14)	100 (129)
Total	89.8 (184)	10.2 (22)	100 (205)
Policy enforced among school workers			
No policy	85.1 (57)	14.9 (10)	100 (67)
Completely enforced	83.8 (31)	16.2 (6)	100 (37)
Partially enforced	93.1 (27)	6.9 (2)	100 (29)
Not all enforced	95.1 (85)	4.9 (4)	100 (89)
Total	89.8 (193)	10.2 (22)	100 (215)
Policy enforced among students			
No policy	90.9 (50)	9.1 (5)	100 (55)
Completely enforced	85.5 (65)	14.5 (11)	100 (76)
Partially enforced	76.9 (10)	23.1 (3)^*^	100 (13)
Not all enforced	97.2 (69)	2.8 (2)	100 (71)
Total	90.2 (194)	9.8 (21)	100 (215)

^*^ *P* < 0.05, ^**^ *P* < 0.001

As shown in (Table 4) in the multiple variable analysis, knowledge about Toombak related health hazards was regressed upon, teaching health issues and preventive practices in schools, this model explained 5.7% of the variance in knowledge (Nagelkerke’s R^2^ .057). School workers who confirmed preventive practices in schools were almost two times more likely than their counterparts to report good knowledge (OR 1.93, CI 1.09–3.43, *p* < 0.05).

**Table 4:** Ever Toombak use and knowledge about health related hazard regressed upon age, attitude of school workers towards their role in Toombak control and enforcement of the policy among students, preventive practices and Teaching health issues

Characteristics	Ever use OR (95% CI)	Knowledge about related health hazard
Age group		
≤ 40 years	1	
> 40 years	4.43 (1.45–13.54)^**^	
Attitude towards their role in Toombak control		
Negative attitude	1	
Positive attitude	0.26(0.08–0.80)^*^	
Policy enforced among students		
No policy	1	
Completely enforced	1.41 (0.41–4.80)	
Partially enforced	3.35 (0.58–19.34)	
Not all enforced	0.41 (0.07–2.40)	
Teaching health issues		
No		1
Yes		1.50 (0.85–2.65)
Preventive practices		
Poor practice		1
Good practice		1.93 (1.09–3.43)^*^

^*^ *P* < 0.05, ^**^ *P* < 0.001

Ever use of Toombak was regressed upon age, enforcement of the policy among students and attitude towards their role in Toombak control and the model explained 23% of the variance (Nagelkerke’s R ^2^ .23). Only age and attitude of school workers towards their role in Toombak control were significant predictors of ever Toombak use. The older group of school workers had almost five times greater likelihood to be Toombak users than their younger counterparts, OR 4.43 (CI 1.45–13.54, *p* < .001). Those with negative attitude had almost four times the likelihood to be Toombak users compared to those with positive attitudes OR 0.26 (CI 0.80–0.86, *p* < .05).

## Discussion

This study was conducted in Sudan and provides baseline information about Toombak use among secondary school workers. Previous studies have focused mostly on dual tobacco (smoke and smokeless) use, while this study focuses only on SLT (Toombak) use [19, 23].

According to the present study, the prevalence of ever use of Toombak was low among the participating school workers, amounting to 9.6%. A previous GSPS study carried out in Sudan in 2009, reported that only 5% of the investigated school workers were using tobacco products other than cigarette (including Toombak and Shisha) [23]. We could, therefore, conclude that the Toombak use among the school workers in this study scores higher than other tobacco products apart from smoking; also when it was compared to other GSPS it was found to be 25.5% in the Central Africa Republic, 6.8% in Saudi Arabia and 17.7% in India [24].

This study has clearly demonstrated that Toombak use is a male dominant habit among school workers in Khartoum State, as all of the users were males. This is different when compared to a study from India. Where, 13.7% of the female versus 35% of the male school workers were SLT users [25]. Male dominance in this study might be considered as information bias because females’ use of Toombak is not socially and culturally acceptable in Sudan. A stigma of females using tobacco products in public is found in low and middle-income countries (e.g. Nigeria and Egypt) as opposed to high-income countries [26–29]. According to WHO, being a male is a strong predictor of tobacco use (48% male versus 12% female) as tobacco use is considered a sign of masculinity among males [30]. This is in accordance with a previous study in Sudan that reported less use of Toombak among females compared to males 1.7% versus 23% [3].

Age has been identified as a strong predictor of Toombak use as it is most prevalent among school workers ≥40 years. This is consistent with previous studies in Sudan. Idris et al. [3] found that the highest prevalence of use was among 70 years old and above, and Ahmed et al. [31] found that 50% of those above 50 years used Toombak compared to 14% of the age group 18–29 years. Similar results have been reported from a study in Bihar, India where older teachers had the greatest tendency to use smoke or smokeless tobacco [32].

Toombak use inside school’s premises on a daily basis was high and this might have a direct influence on students’ use of tobacco and may cause a detrimental effect to the school workers’ function as a role model for students as was shown by other studies [33–35]. In Sudan, Elamin et al. [19] reported that Toombak use by school workers’ inside the school premises can influence students’ smoking and Toombak use.

Schools expected to be a favourable place to promote health and also to protect the students from tobacco exposure by effective school tobacco control policies [36]. Low effectiveness of tobacco policies inside school premises (Table 3), however, is a global problem which has been shown in studies from other countries e.g. India and Bangladesh, where school policy was reported to have poor enforcement [24, 37]. Nevertheless, some studies have emphasized the effectiveness of some components of the policy in preventing students from tobacco use [38, 39], while others, Coppo et al. [40], have questioned the effectiveness of the school tobacco policies where they found no difference between tobacco use prevalence among schools where policy is effective or not effective. Coppo et al. [40], concluded that, “the absence of reliable evidence for the effectiveness of school tobacco policy is a concern for public health”.

In this study, reduced knowledge of the health hazards of Toombak use among school workers is a common problem, especially in Africa as most of the school workers demonstrate low awareness of the health consequences of tobacco products use [19, 41]. Being unaware of the health risks of Toombak use might reduce participation in prevention activities and thus provide less support to tobacco control policy among an important group of school workers [23, 41, 42].

Knowledge in this study is consistently significant among those who reported participation in preventive practices in schools. This is in accordance with Agaku et al. [41] who reported that knowledge of school workers increased among those who were actively involved in preventive activities inside schools.

The majority of the participants, more females than males, showed a positive attitude towards their role in Toombak control (Table 2). This might be explained by the fact that females showed better knowledge and this also might explain the finding that all the Toombak users were males. This might reflect the role of knowledge in forming people’s attitude according to behavioural models that suggest the importance of knowledge and attitude for behavioural change [43]. School workers whom non- users demonstrated more positive attitude towards their role in Toombak control than ever users and that is found in other professions e.g. health professionals where social habits like, tobacco use, can negatively affect the professionals’ behaviours in advising and guiding others as role models. Usually, they see the habit of tobacco use as a social habit rather than health problem [44, 45]. This is in agreement with previous GSPS study in Sudan where overwhelming majority showed a negative attitude towards Toombak use [23].

However; this study has its limitations, as the findings are based on self-reporting that might be subjected to information bias (social desirability), thus school workers may either have under- or over-reported their responses to the questionnaire. Also, the participation was voluntary; it may not be subjected to selection bias as all school workers accepted to participate in the study. In addition, the study sample of school workers might not be representative of all school workers in Khartoum state.

The Alpha values (Cronbach’s α) reported in this study were lower than the expected rule of thumb (0.7), this may affect the reliability of some questions. However, some factors may affect the test as the number of items, the type of variables [46]. Thus the results, although reflect a realistic picture of the current situation, should be interpreted with caution.

## Conclusions

Although the prevalence of Toombak use among the school workers investigated was low, Toombak use was associated with poor knowledge, negative attitude towards their role in Toombak control and poor preventive practice. Thus, school workers who use Toombak may be less motivated for the prevention of the major health problems caused by Toombak use and less suitable as role models for their students in tobacco prevention. On the other hand, school workers who were engaged in preventive practices in schools presented with good knowledge which, in turn, may empower their positive attitude towards their role in Toombak control.

## Abbreviations

FCTC: Framework convention on tobacco control
GSPS: Global School Personnel Survey
GTTS: Global Tobacco Surveillance System
SLT: Smokeless tobacco
TSNAs: Tobacco specific N-nitrosamines
WHO: World Health Organization
